# Risk factors for the evolutionary emergence of pathogens

**DOI:** 10.1098/rsif.2010.0123

**Published:** 2010-04-21

**Authors:** H. K. Alexander, T. Day

**Affiliations:** Department of Mathematics and Statistics, Queen's University, Kingston, Ontario, Canada K7L 3N6

**Keywords:** evolutionary epidemiology, emerging infectious diseases, multi-type branching process, mutation, contact network, mathematical model

## Abstract

Recent outbreaks of novel infectious diseases (e.g. SARS, influenza H1N1) have highlighted the threat of cross-species pathogen transmission. When first introduced to a population, a pathogen is often poorly adapted to its new host and must evolve in order to escape extinction. Theoretical arguments and empirical studies have suggested various factors to explain why some pathogens emerge and others do not, including host contact structure, pathogen adaptive pathways and mutation rates. Using a multi-type branching process, we model the spread of an introduced pathogen evolving through several strains. Extending previous models, we use a network-based approach to separate host contact patterns from pathogen transmissibility. We also allow for arbitrary adaptive pathways. These generalizations lead to novel predictions regarding the impact of hypothesized risk factors. Pathogen fitness depends on the host population in which it circulates, and the ‘riskiest’ contact distribution and adaptive pathway depend on initial transmissibility. Emergence probability is sensitive to mutation probabilities and number of adaptive steps required, with the possibility of large adaptive steps (e.g. simultaneous point mutations or recombination) having a dramatic effect. In most situations, increasing overall mutation probability increases the risk of emergence; however, notable exceptions arise when deleterious mutations are available.

## 1. Introduction

The recent outbreaks of SARS coronavirus and avian and swine influenza strains have highlighted the importance of pathogen cross-species transmission, and subsequent evolutionary adaption, in the emergence of new diseases (Woolhouse *et al.* 2005; Dennehy 2009). Such ‘species jumps’ have been proposed as a major source of novel pathogen introductions (Woolhouse *et al.* 2005; Day *et al.* 2006; Kuiken *et al.* 2006), and indeed many emerging human diseases are zoonotic (Cleaveland *et al.* 2001; Taylor *et al.* 2001). Typically, these pathogens are initially poorly adapted to humans because of physiological differences between species (Kuiken *et al.* 2006). Thus, significant spread of these pathogens within the human population often requires evolutionary adaptation (Dennehy 2009).

A number of risk factors have been proposed to explain why some pathogens emerge and others do not. These include the breadth of the pathogen's host range (Cleaveland *et al.* 2001; Taylor *et al.* 2001; Woolhouse & Gaunt 2007), susceptibility of the host (Woolhouse *et al.* 2005; Woolhouse & Gaunt 2007), contact patterns in the host population (Woolhouse *et al.* 2005; Kuiken *et al.* 2006; Woolhouse & Gaunt 2007; Dennehy 2009), the mechanism(s) of pathogen adaptation (e.g. whether recombination is possible; Woolhouse *et al.* 2005), pathogen taxonomic classification (Cleaveland *et al.* 2001; Taylor *et al.* 2001), pathogen generation time (Cleaveland *et al.* 2001; Taylor *et al.* 2001) or growth rate (Dennehy 2009) and pathogen mutability (Cleaveland *et al.* 2001; Taylor *et al.* 2001; Woolhouse *et al.* 2005; Dennehy 2009). Indeed, empirical studies have revealed that some of these factors are often associated with emerging diseases (Cleaveland *et al.* 2001; Taylor *et al.* 2001; Woolhouse & Gaunt 2007). In particular, viruses and protozoa display a relatively high propensity for being involved in newly emerging human diseases (Cleaveland *et al.* 2001; Taylor *et al.* 2001), and the degree of transmissibility between members of the new host species also appears to be a risk factor where data are available (Taylor *et al.* 2001).

The association between transmissibility and likelihood of emergence is, perhaps, not surprising. Any factor that increases the expected number of transmitted infections, such as higher initial transmissibility between members of the new host species, means that the pathogen can circulate for longer after the cross-species jump, and thus has greater potential for ultimate evolutionary adaptation (Antia *et al.* 2003). For the same reason, patterns of contact among hosts should also play an important role in disease emergence, because some contact structures ought to lead to greater scope for transmission than others. To date, however, the most ‘risky’ patterns of host contact are not known.

The association between pathogen taxonomic classification and likelihood of emergence is less easy to explain. It has been suggested that the high incidence of emergence among viruses may be attributed, at least partially, to their high mutation rates (particularly in RNA viruses). A high mutation rate might lead to a greater likelihood of the appropriate adaptive mutations occurring, implying greater evolutionary potential (Cleaveland *et al.* 2001; Woolhouse *et al.* 2005). On the other hand, most mutations are deleterious, and a high mutation rate could thereby increase the chance of pathogen extinction (Dennehy 2009). Indeed, this propensity for extinction might be exploited clinically by drug-induced ‘lethal mutagenesis’ (Anderson *et al.* 2004; Clementi 2008). Thus, it remains unclear whether higher mutation rates do indeed lead to a higher likelihood of evolutionary emergence, or whether other processes are required to explain the empirical patterns.

In this paper, we investigate the above factors through mathematical modelling. We ask two main questions: (i) what types of host contact structure lead to the greatest risk of evolutionary emergence? and (ii) how do patterns of mutation affect the risk of evolutionary emergence? We address these questions using a branching process model that tracks the number of infected hosts as a newly introduced pathogen spreads and evolves. There have been two main approaches to using such models in the epidemiological literature, a network-based approach and a phenomenological approach. The former explicitly considers the patterns of contact among individuals in the population and the probability of transmission through any given contact (e.g. Brauer 2008), but has not, to date, allowed for evolution. The latter assumes that the population is well mixed and in some cases allows for evolution, but has not explicitly modelled the contact patterns among individuals (e.g. Antia *et al.* 2003; Lloyd-Smith *et al.* 2005; Day *et al.* 2006; Yates *et al.* 2006; Reluga *et al.* 2007). In order to address the above questions, we place these two approaches within a common framework, generalizing to allow for arbitrary contact patterns and arbitrary pathways of evolutionary adaptation.

## 2. A unified modelling framework

In order to develop a unified modelling framework that encompasses both the network-based and phenomenological approaches, it is useful first to focus on single-strain models that ignore evolution.

### 2.1. Single-strain models

We model in discrete time, using a Galton–Watson process. To define the branching process, we require a distribution for the number of ‘offspring’ produced by each individual. In the present context, infected hosts are the individuals of interest, and an ‘offspring’ is viewed as a contact to whom the infection is transmitted. The network-based approach and the phenomenological approach arrive at this offspring distribution in different ways. The network-based approach builds up the offspring distribution from underlying assumptions about the processes of host–host contact and pathogen transmission. The phenomenological approach, on the other hand, simply specifies this offspring distribution directly, without explicit consideration of the underlying contact and transmission processes through which it might arise.

The network-based approach represents the host population by a graph: each vertex or node corresponds to an individual, and an edge between two nodes signifies that the two individuals are acquaintances, i.e. can contact and potentially transmit disease to one another. We assume that this network is static, and restrict attention to random graphs in the limit of infinite population size. In this situation, the network becomes a tree graph, with the number of ‘branches’ from each node drawn independently from the arbitrary degree, or contact, distribution (Trapman 2007 and references therein). This restriction is applied for mathematical tractability, but neglects certain realistic network properties such as loops and clustering; we return to this issue in §4.3. We refer to the individual(s) initially infected by a source outside the population as ‘generation 0’ infectives. All individuals infected by a generation *n* infective (*n* = 0, 1, …) are considered generation *n* + 1 infectives, and ‘later-generation’ infectives (i.e. generation ≥ 1) receive their infections from other individuals within the population.

Following the derivation presented by Brauer (2008), we suppose that each node has a degree distribution {*p*_*d*_}, described by the probability generating function (PGF)

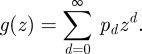

Furthermore, if we randomly choose an edge and follow it to a node, the excess degree of that node (number of other edges emanating out) has distribution {*p̃*_*d*_} given by the PGF

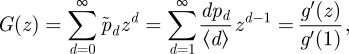

where 〈*d*〉 = *g*′(1) denotes the expected value of degree *d*.

An infective is assumed to transmit infection to each still-susceptible contact *independently* with probability *T*, called the transmissibility, taken to be a constant. That is, if *d* is the number of still-susceptible contacts, then the number of infections transmitted is distributed as Binomial(*d*, *T*). For the initial infective, who has been infected by a source outside the population, all contacts (given by the degree) remain susceptible. Thus, the PGF *γ*(*s*) for the number of infections transmitted by a randomly chosen initial infective is (Brauer 2008)

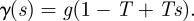

A later-generation infective has received the infection from one of its contacts, i.e. it has been arrived at by following a randomly chosen edge from another node. Hence, the number of still-susceptible contacts is determined from the excess degree distribution. (We implicitly assume that an individual can only be infected once, as in an SI- or SIR-type disease.) Thus, the PGF *Γ*(*s*) for the number of infections transmitted by a later-generation infective is

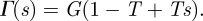

The basic reproductive number, *R*_0_, can then be defined as the mean number of infections transmitted by one typical (later-generation) infective:

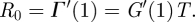

In this way, we can see how the disease's reproductive number in the network-based approach is composed of the underlying processes of host–host contact (represented by *G*′(1)) and pathogen transmission (represented by *T*). *R*_0_ is an appropriate measure of the fitness of a single strain of pathogen.

The phenomenological approach (e.g. Antia *et al.* 2003; Lloyd-Smith *et al.* 2005; Day *et al.* 2006; Yates *et al.* 2006; Reluga *et al.* 2007) does not build up the process of disease spread from a description of host contacts and pathogen transmission. Rather, it directly specifies a distribution of number of new infections, *X*, produced by an infected individual. The approach typically uses a Poisson distribution of infections, implicitly assuming homogeneous mixing in the population, but sometimes incorporates individual heterogeneity by allowing the mean of the distribution itself to be a random variable. For example, Lloyd-Smith *et al*. (2005) generally draw the mean, *ν*, from a Gamma distribution with mean *R*_0_ and dispersion parameter *β*; then *X* has a negative binomial distribution with mean *R*_0_ and dispersion *β*. As special cases, *β* = 1 corresponds to *ν* ∼ Exp(*R*_0_) and *X* ∼ Geometric(*R*_0_), while *β* → ∞ corresponds to *ν* = *R*_0_ (no individual variation) and *X* ∼ Poisson(*R*_0_). In any case, the key difference from the network-based approach is that *Γ*(*s*) is effectively specified directly. As before, one then defines the basic reproductive number as *R*_0_ = *Γ*′(1). Notice, however, that unlike the network-based approach, no distinction is made here between generation 0 infectives (i.e. those who receive the infection from an outside source) and later-generation infectives. (In special cases of the network model, all generations are in fact equivalent; see appendix A.2.) If we consider only later-generation infectives, the phenomenological approach can be obtained via the network-based approach, provided that the contact distribution in the latter is chosen to obtain the same end result for the offspring distribution (appendix A.3).

**Figure 1.**
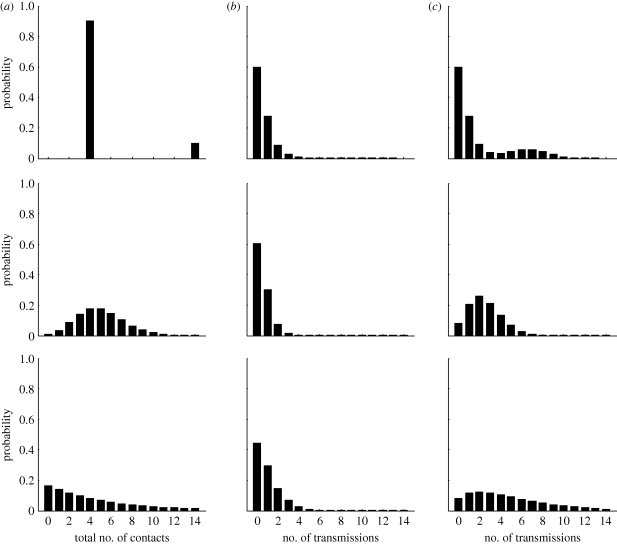
(*a*) Examples of contact distributions, described by the PGF *g*(*z*), along with the corresponding offspring distributions, described by the PGF *Γ*(*s*), for transmissibility (*b*) *T* = 0.1 and (*c*) *T* = 0.5. Contact distributions, all with mean 5, are: top row, mixed deterministic with 10% superspreaders; middle row, Poisson; bottom row, negative binomial with dispersion *β* = 1 (i.e. geometric).

For example, if the contact distribution is such that the number of still-susceptible contacts has a negative binomial distribution with mean *λ* and dispersion *β* (appendix A.1), i.e. *G*(*z*) = (1 + (*λ*/*β*)(1 − *z*))^−*β*^ (Lloyd-Smith *et al.* 2005), then the offspring distribution will also be negative binomial with mean *λT* and dispersion *β*: *Γ*(*s*) = *G*(1 − *T* + *Ts*) = (1 + (*λT*/*β*) (1 − *s*))^−*β*^. Since *R*_0_ = *λT*, this gives exact correspondence with the phenomenological approach of Lloyd-Smith *et al.* (2005) described above. Other examples of contact distributions and the resulting offspring distributions are described in appendix B.1 and illustrated in figure 1.

Regardless of which approach is used to obtain the offspring distribution, the *extinction probability* *q* of the process—that is, the probability that the disease outbreak eventually ends, infecting only a finite number of people—is the smallest non-negative solution of the equation *Γ*(*s*) = *s*. The epidemic is guaranteed to go extinct if *R*_0_ ≤ 1, but persists with non-zero probability if *R*_0_ > 1 (Allen 2003). Recall, however, that the network-based approach also distinguishes the generation 0 infective from all others, and if this initial infective is chosen uniformly at random, then the overall extinction probability in that approach is *g*(1 − *T* + *Tq*) ≡ *γ*(*q*) (Brauer 2008).

### 2.2. Multiple strain model

A phenomenological approach has also been used to explore how pathogen evolution affects disease emergence by accounting for multiple pathogen strains (Antia *et al.* 2003; André & Day 2005; Day *et al.* 2006; Yates *et al.* 2006; Reluga *et al.* 2007). These models typically assume a Poisson distribution of infectious contacts, which, interestingly, has been found usually to be a poor fit to epidemiological data (Lloyd-Smith *et al.* 2005). Mathematically similar models have also been used to model evolution and ‘escape’ in a population of replicating individuals (Iwasa *et al.* 2003, 2004; Serra & Haccou 2007). In the most general of these, Serra & Haccou (2007) outline the model for an arbitrary offspring distribution as well as arbitrary mutation scheme and fitness landscape. However, to date, such generalizations and their implications have not been explored in the context of evolutionary epidemiology, nor have underlying mechanisms contributing to the offspring distribution been considered separately. One can readily extend the network-based framework outlined in §2.1 to investigate these questions.

We account for pathogen evolution using a multi-type branching process. An individual's type *i* (*i* = 1, … , *m*) denotes which one of the *m* possible pathogen strains is infecting that host. We allow for arbitrary contact distribution and assume that the contact network is determined by the host, independently of the pathogen strain. Thus, as before, degree is described by the PGF *g*(*z*) and the excess degree by *G*(*z*) for every individual, regardless of type. We retain the assumption that transmissions occur independently to each contact, and further assume that transmissibility *T* is strain-specific, denoted *T*_*i*_ for strain *i*. Thus, the probability of making an infectious contact depends only on the current infective's strain type. However, a different strain may be transmitted as a result of pathogen mutation. Specifically, given that a type *i* infective makes an infectious contact, strain *j* is transmitted with probability *μ*_*ij*_ (∑_*j*_ *μ*_*ij*_ = 1, 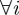). We call the *m* × *m* matrix *U* = [*μ*_*ij*_] the ‘mutation matrix’, representing the mutational pathway(s) allowed in the evolution of the pathogen and their probabilities.

We use the word ‘mutation’ loosely to mean any processes resulting in a change in the strain identified as a host's type. These processes actually occur *within* individual hosts, but we account for this in a phenomenological way. The most accurate interpretation of our approach would be that a host currently infected with strain *i* transmits only strain *i*, but that each infective produced in the next generation has probability *μ*_*ij*_ of converting to strain *j* over the course of infection, before any further transmission occurs. In this approach, *μ*_*ij*_ is an ‘effective conversion rate’ from strain *i* to strain *j* within one host, summarizing the results of within-host dynamics (§4.3). This is a common type of simplification involving a separation of the time scales on which within- and between-host processes occur. Antia *et al*. (2003) mention the possibility of strain conversion owing to mutation within a host, and André & Day (2005) explicitly model this in a phenomenological way; however, neither discuss the above considerations in any detail.

Returning to our multi-type process, the number of transmissions of each type made by a type *i* infective with *d* susceptible contacts now has distribution Multinomial(*d*; 1− *T*_*i*_, *T*_*i*_*U*_*i*·_), where *U*_*i*·_ is the *i*th row of *U* and the probability vector is given in the order: no transmission, transmit strain 1, … , transmit strain *m*. The corresponding PGF for the number of transmitted infections of each type, (*X*_1_ , … , *X*_*m*_), given *d* susceptible contacts, is

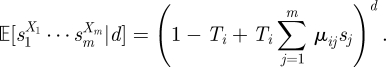

Thus, extending the notation of §2.1, the PGF for number of infections transmitted by an initial infective of type *i* is

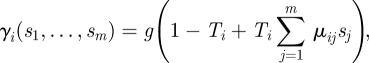

and for a later generation infective, the PGF is

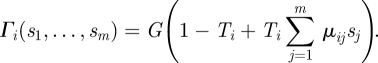

Derivations of these PGFs appear in appendix C.

The probability of extinction starting from one later-generation infective of type *i*, denoted *q*_*i*_, is obtained as the smallest non-negative root of the equation *q*_*i*_ = *Γ*_i_(*q*_1_, … , *q*_*m*_), solved simultaneously for all *i* (appendix D). More compactly, we can write this as a vector fixed-point equation: 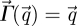, where 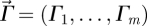 and 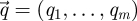. Starting from a generation 0 infective of type *i*, chosen uniformly at random, the overall probability of extinction is then given by *g*(1 − *T*_*i*_ + *T*_*i*_ ∑ _*j*=1_^*m*^ *μ*_*ij*_*q*_*j*_) ≡ *γ*_*i*_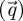 (appendix C). If we account for strain conversion as per the above interpretation, the only adjustment we must make in our calculations, assuming that strain *i* is introduced to a randomly chosen individual, is to allow the initial infective to be type *j* with probability *μ*_*ij*_. We generally assume that strain 1 is, by definition, initially introduced to the host population.

In what follows, it is sometimes useful to refer to the ‘basic reproductive number of strain *i*’, denoted *R*_0,*i*_ and defined as the expected *total* number of infectious contacts made by a typical (later-generation) type *i* infective. Since this number is distributed as Binomial(*d*, *T*_*i*_), we have

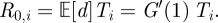

If strain *i* were the only strain present, with no possibility of mutation, *R*_0,*i*_ would be the value of its basic reproductive number in the single-strain model.

We define emergence as the situation in which the pathogen escapes extinction, which necessarily requires evolution to a strain having *R*_0,*i*_ > 1. Probability of emergence is thus the complement of probability of extinction. We present numerical results for the probability of emergence beginning from one later-generation infective of type 1, 1 − *q*_1_, but these results may be extended to account for generation 0 and/or type conversion as described above. Note that the branching process may be either indecomposable or decomposable, depending on the mutational scheme. Thus, if the epidemic persists, the complement of strains that will be present must be treated on a case-by-case basis (appendix D).

Our derivation so far is quite general, imposing no *a priori* restrictions on the choices of contact distribution, transmissibilities and mutation probabilities. However, in the numerical results to follow, we limit ourselves to specific examples.

*Contact distributions*. We consider the following contact distributions, each with mean *λ* (see also appendix B.1).

— *Deterministic*. Every individual has exactly *λ* contacts.
— *Mixed deterministic*. There are *n* types of individuals, where the *k*th type occurs in proportion *p*_*k*_ and has exactly *λ*_*k*_ contacts.
— *Poisson*.
— *Mixed Poisson*. There are *n* types of individuals, where the *k*th type occurs in proportion *p*_*k*_ and has a Poisson(*λ*_*k*_) distribution of contacts.
— *Negative binomial*, with dispersion *β*.

*Mutational schemes*. We consider two broad types of mutational schemes: ‘linear’ and ‘hub-and-spoke’ (see also appendix B.2). In all cases, we assume that strain *m* is well adapted to the host; that is, *T*_*m*_ is chosen such that *R*_0,*m*_ > 1. On the other hand, any other strain *i* is poorly adapted to the host (*R*_0,*i*_ < 1) unless otherwise specified.

Linear mutational schemes represent single directions through strain space, where strains 2, … , *m* − 1 are intermediates between strains 1 and *m*. We consider the following possibilities.

— *One-step irreversible*. The pathogen must acquire *m* − 1 point mutations, one at a time and in a fixed order. Thus, mutation occurs only from strain *i* to *i* + 1. This scheme is presented by Antia *et al.* (2003).
— *Multi-step irreversible*. Again the pathogen must acquire *m* − 1 point mutations in a fixed order, but now possibly simultaneously, implying that mutations can occur from strain *i* to any strain *j* > *i*, though with diminishing probabilities. Essentially, higher order terms are being included where they were neglected in the previous scheme. This scheme is presented by Gokhale *et al.* (2009), along with biological examples.
— *Interchangeable and irreversible*. This scheme, also presented by Gokhale *et al.* (2009), is similar to the preceding one, but allows the point mutations to be acquired in arbitrary order, introducing a combinatorial aspect to the probabilities. Thus, there are multiple evolutionary pathways between the first and last strains, with strain *i* in our model representing any ‘real’ strain having *i* − 1 of the required mutations. Our model thus implicitly assumes that any collection of *i* − 1 mutations yields the same transmissibility, *T*_*i*_.
— *Point mutation and recombination*. One-step point mutation occurs as in scheme 1, but we simplistically model an additional mechanism of more extensive genetic change (perhaps recombination or reassortment; Kuiken *et al.* 2006) by allowing any strain *i* to adapt directly to strain *m* with a probability not tied to that of point mutation. We neglect the chance of simultaneous mutational events, and all are irreversible.
— *One-step reversible*. In a modification of scheme 1, mutation can occur from strain *i* to either *i* + 1 or *i* − 1, representing both forward and reverse point mutation (again neglecting the chance of simultaneous mutations).

One-step irreversible mutations, coupled with a Poisson(*λ*) distribution of contacts, corresponds to the model of Antia *et al.* (2003), where *R*_0,*i*_ = *λT*_*i*_.

Hub-and-spoke mutational schemes represent multiple distinct pathways through strain space. After strain 1 is introduced, mutation may proceed from this ‘hub’ along a number of different ‘spokes’ (directions in strain space). Though still numbered sequentially for convenience, strains 2, … , *m* − 1 no longer represent intermediates on the path to strain *m*. For simplicity, in our results we consider only paths of length 1 (i.e. strains 2, … , *m* represent *m* − 1 distinct pathways), with equal probability of proceeding along any path. However, this set-up could obviously be extended to incorporate pathways of various lengths and mutation probabilities. We consider two possible scenarios.

— *One-step irreversible*.
— *One-step reversible*.

Finally, we must specify the transmissibility of each strain, 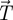, particularly how these values relate to one another. For a given contact distribution, defining 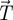 effectively determines the pathogen's fitness landscape. For linear mutation schemes, we will typically use the ‘jackpot model’ (Antia *et al.* 2003), in which strains 1 through *m* − 1 have identical transmissibility, and hence the same value of *R*_0,*i*_ < 1. Other biologically interesting possibilities include a fitness valley, where intermediate strains have lower fitness than strain 1, or an additive model, where fitness increases linearly with strain number (Antia *et al.* 2003). For hub-and-spoke schemes, we take *T*_2_ , … , *T*_*m*−1_ less than *T*_1_, representing deleterious mutations, while only *T*_*m*_ is greater than *T*_1_, representing a beneficial mutation.

## 3. Results

### 3.1. Criticality of the process

The basic reproductive number, *R*_0_, is widely used as a predictor of when a disease has epidemic potential, as well as a descriptor of disease spread (Anderson & May 1991; Allen 2003; Brauer 2008). In branching process models, the disease has a positive probability of emergence if, and only if, *R*_0_ > 1. Other aspects of the offspring distribution affect the probability of emergence as well, and thus it is common to compare the probability of emergence across different distributions at the same value of *R*_0_ (Lloyd-Smith *et al.* 2005; Brauer 2008).

As clearly illustrated in the network-based approach, however, *R*_0_ is a composite quantity that is influenced by the combined processes of host-to-host contacts and disease transmission (Meyers *et al.* 2005; Brauer 2008). Once we decompose the process of disease spread into these mechanistic components, other comparisons suggest themselves. In particular, given that *R*_0_ itself is a critical threshold quantity for disease spread, it is natural to ask how this quantity changes across different contact distributions that have the same mean number of contacts and the same disease transmissibility. We might also ask how probability of emergence changes as a function of *T* rather than *R*_0_. Meyers *et al.* (2005) likewise argue for a focus on transmissibility, rather than *R*_0_, from the perspective of making reliable public health predictions across different host subpopulations.

Insight into the comparison between contact distributions can be gained by rewriting the equation *R*_0_ = *G*′(1)*T*. Using the relationship *G*(*z*) = *g*′(*z*)/*g*′(1) gives *R*_0_ = (*g*″(1)/*g*′(1))*T*. Then substituting *g*″(1) = *σ*^2^ − *g*′(1) + (*g*′(1))^2^, where *σ*^2^ is the variance of the contact distribution, yields

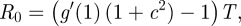

where *c* = *σ*/*g*′(1) is the coefficient of variation of the contact distribution. For a given transmissibility and mean number of contacts, the contact distribution that maximizes variance thus maximizes *R*_0_. The above equation is essentially the same as the expression for *R*_0_ given by Meyers *et al.* (2005), although there it is not rewritten in terms of *c*, and is similar to the expression for *R*_0_ used in deterministic network models (May & Lloyd 2001).

Analogously for a multi-type process, the expectation or mean matrix *M* = [*a*_*ij*_], where 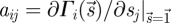 is the expected number of type *j* progeny of one type *i* individual, is crucial in determining criticality of the process (Harris 1963; Allen 2003; Haccou *et al.* 2005). If the dominant eigenvalue of *M* is less than or equal to one, extinction is certain; otherwise, there is a positive probability of non-extinction and the process is classified as ‘supercritical’ (Harris 1963; Haccou *et al.* 2005). This threshold result holds whether the process is indecomposable or decomposable (see appendix D). The dominant eigenvalue of *M* is also known as the population-wide basic reproductive number.

In our multiple strain model, with contact distribution given by PGF *g*(*z*) and mutation probabilities given by matrix *U* = [*μ*_*ij*_], we have *a*_*ij*_ = *G*′(1)*T*_*i*_ *μ*_*ij*_ = (*g*′(1)(1 + c^2^) − 1)*T*_*i*_*μ*_*ij*_. We can thus express the mean matrix as

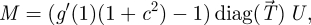

emphasizing the dependence of *M*—and hence its dominant eigenvalue—on the mean and variance of the contact distribution, the transmissibility of each strain and the mutation scheme.

### 3.2. Impact of contact distribution and transmissibility on emergence

**Figure 2.**
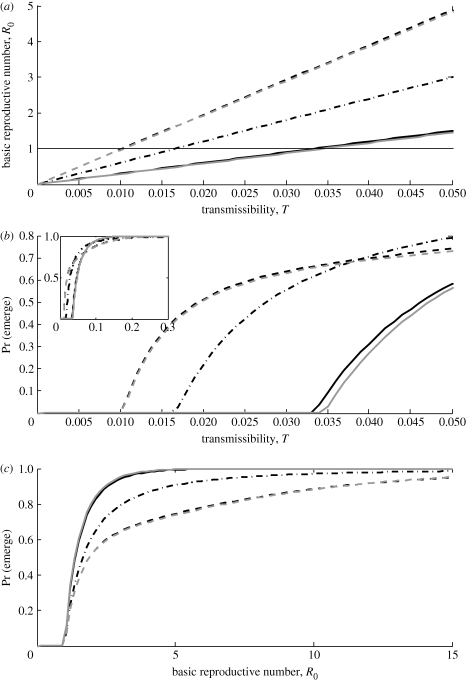
The relationships among transmissibility, basic reproductive number and probability of emergence, for a single strain of pathogen and various host contact distributions: Poisson (solid black), mixed Poisson (dashed black), negative binomial with dispersion 1 (dot-dash black), deterministic (solid grey) and mixed deterministic (dashed grey). Mean number of contacts is fixed at 30. For mixed distributions, there are two types of hosts, with 90% of the population having on average 15 contacts and 10% having on average 165 contacts.

Figure 2 illustrates, for a single strain, the impact of contact distribution on the basic reproductive number, *R*_0_, and on the probability of emergence starting from one later-generation infective, 1 − *q*. Figure 2*a* shows how *R*_0_ increases with transmissibility *T*: in all cases, this increase is linear, but at a different rate for each contact distribution depending on the variance in contacts. Figure 2*b* plots the probability of emergence versus *T* for each contact distribution. This probability becomes non-zero at the point where *R*_0_ passes the critical value of one, which occurs at a different value of *T* for each distribution (as illustrated in figure 2*a*). Thus, at low values of *T*, the disease can persist in some host contact structures but not others, despite the fact that they all have the same mean number of contacts. As *T* increases, however, the probability of emergence increases at different rates for different contact structures, such that the ordering is not preserved. This observation is highlighted in the inset, showing emergence probability over an extended range of *T*. In contrast, the ordering is consistent in figure 2*c*, which plots the probability of emergence versus *R*_0_, comparable to Brauer's presentation (Brauer 2008). The same value of *R*_0_ has been achieved for each contact distribution by varying *T* to compensate. We can think of the middle plot as showing the net result of opposing influences illustrated in figure 2*a*,*c*. Similar considerations and results on the impact of contact distribution and transmissibility apply to the multiple strain case.

### 3.3. Impact of mutation scheme on criticality

**Figure 3.**
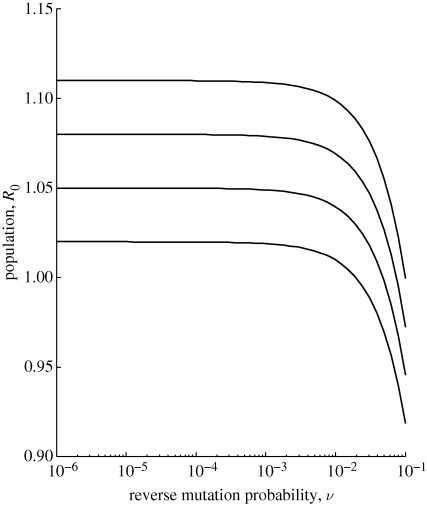
As reverse mutation probability (*ν*) increases, the dominant eigenvalue of the mean matrix (population *R*_0_) decreases, changing the criticality of the branching process. Results are illustrated for *m* = 2 strains, with *T*_1_ = 0.01 and various values of *T*_2_ spaced linearly from 0.034 (bottom curve) to 0.037 (top curve). Contact distribution is Poisson with mean 30 and forward mutation probability is *μ* = 0.01.

When there are multiple strains of pathogen, the pathways of mutation among them may also affect the threshold parameter. In the case of irreversible mutation, we can number strains such that *M* is a triangular matrix, with its eigenvalues given simply by the entries on the main diagonal. Assuming the final strain is the best adapted, the dominant eigenvalue is then *R*_0,*m*_ = *G*′(1)*T*_*m*_. Hence, criticality is independent of the precise details of a uni-directional mutation scheme. By contrast, a scheme that allows both forward and reverse mutation can affect criticality. Figure 3 illustrates how the population *R*_0_ (dominant eigenvalue of *M*) changes with the probability of reverse mutation (*ν*) in the linear one-step reversible scheme; similar considerations apply to the hub-and-spoke one-step reversible scheme as well. A high rate of reverse mutation from a well-adapted to a poorly adapted strain can tip the branching process from supercritical to subcritical. Population *R*_0_ increases almost linearly with *T*_*m*_, but is relatively insensitive to number of strains (*m*), earlier strain transmissibility (*T*_1_) and forward mutation probability (*μ*). This is because population *R*_0_ is very close to *a*_*mm*_ = *G*′(1)*T*_*m*_(1 − *ν*), but slightly influenced by contributions from small populations of other (poorly adapted) strains that persist asymptotically if the process escapes extinction.

### 3.4. Impact of mutation scheme on probability of emergence

We can compare not only the qualitative result of criticality, but also the quantitative probability of emergence across various supercritical branching processes. To obtain this probability, 1 − *q*_1_, we compute the extinction probability 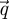 numerically, through fixed-point iterations of the offspring distribution PGF, 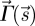 (§2.2). We initiate an iteration from a point 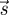 with *s*_*i*_ < 1, 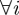, to ensure convergence to the appropriate fixed point (see appendix D for details), and continue until the difference between successive iterations, in max norm, is less than a specified tolerance, taken to be 10^−12^ or 10^−16^ in the figures we present.

**Figure 4.**
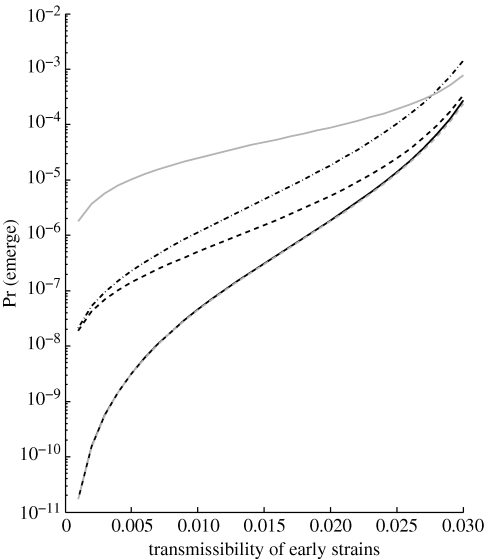
Probability of emergence (1 − *q*_1_) for various linear mutation schemes: one-step irreversible (solid black), multi-step irreversible (dashed black), interchangeable and irreversible (dot-dash black), point mutation and recombination (solid grey) and one-step reversible (dashed grey). Contact distribution is Poisson with mean 30. We use *m* = 4 strains and the jackpot model of evolution, with transmissibility of the final strain set to 0.05. Forward mutation probability is *μ* = 0.01 and, where applicable, reverse mutation probability is *ν* = 0.01 and jump-to-*m* probability is *ρ* = 0.0001.

Figure 4 illustrates the probability of emergence across various linear mutation schemes with the same contact distribution. Here, *T*_*m*_ is far from the critical threshold, and reverse mutation, even at a high rate equal to that of forward mutation, makes a negligible impact on the probability of emergence. This agrees with Sagitov and Serra's result that reverse mutation is negligible in a similar model with an arbitrary offspring distribution (Sagitov & Serra 2009), and with the neglect of pathways to the escape mutant exceeding the minimum length in the work of Iwasa *et al.* (2003, 2004). On the other hand, results are highly sensitive to the path of forward mutation that is assumed to be possible. Allowing jumps directly to strain *m* tends to make a particularly large difference, with this effect most pronounced when early strains have very low transmissibility. Simultaneous point mutations, though extremely rare, also make a significant contribution to the probability of emergence. In general, similar trends were observed for larger *T*_*m*_, with the probability of emergence scaled up but the relationships among mutation schemes the same. Probability of emergence reaches a plateau, presumably at the probability of the well-adapted strain *m* ever appearing, as *T*_*m*_ becomes very large (results not shown).

We can also consider the impact of mutation scheme when intermediate strain fitness (as determined by transmissibility for fixed contact structure) varies. Although we have typically used the jackpot model, one might also consider other choices of transmissibilities. As one would expect, the higher the intermediate strain fitness, the higher the probability of emergence as observed by Antia *et al.* (2003). Now that we have generalized the mutation scheme, we can investigate whether this effect is more pronounced for some schemes than for others. For instance, a decrease in intermediate strain transmissibility (creating a fitness valley) hurts the pathogen's chance of emergence more if only one-step forward mutation is possible, as opposed to a jump directly to the final strain through recombination (results not shown). This is in agreement with an observation by Iwasa *et al.* (2004).

### 3.5. Impact of mutation probabilities and number of strains

For any given mutation scheme, we can gain greater insight into the effect of mutation probabilities and number of strains by estimating the probability of emergence analytically. As other authors (Antia *et al.* 2003; Iwasa *et al.* 2003, 2004; Serra & Haccou 2007; Sagitov & Serra 2009) have considered analytical approximations for this model (or special cases of it) in detail, we keep our remarks brief.

Using an intuitive argument, Antia *et al.* derived the following approximation for the probability of evolution to strain *m* (starting from strain 1) in the case of a Poisson-distributed number of infectious transmissions and one-step irreversible mutation (Antia *et al.* 2003):

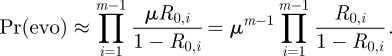

**Figure 5.**
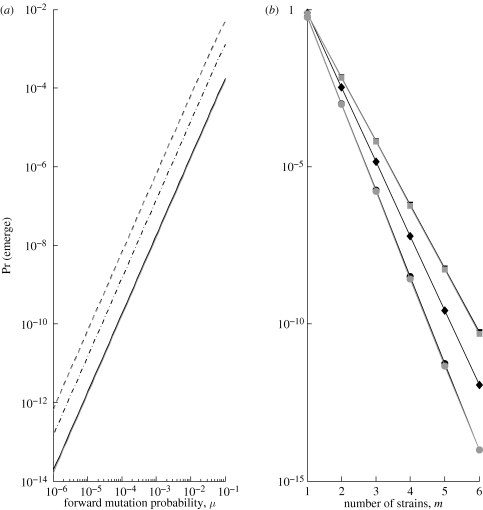
Probability of emergence (1 − *q*_1_) as a function of (*a*) forward mutation probability (*μ*) and (*b*) number of strains (*m*) in the one-step irreversible scheme, for various contact distributions: Poisson (solid black/black circles), mixed Poisson (dashed black/black squares), negative binomial with dispersion 1 (dot-dash black/black diamonds), deterministic (solid grey/grey circles) and mixed deterministic (dashed grey/grey squares). Contact distribution parameters are set as in figure 2. We use the jackpot model of evolution: *T*_*i*_ = 0.005, 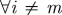, and *T*_*m*_ = 0.05. (*a*) Number of strains is fixed at *m* = 3; (*b*) forward mutation probability is fixed at *μ* = 0.01.

This approximation, which holds for *μ* ≪ 1 and *R*_0,*i*_ not too close to 1, makes it clear that probability of emergence, which is proportional to the probability of evolution in this estimation, is expected to scale ∼*μ*^*m*−1^ (Antia *et al.* 2003). The derivation in fact proceeds without reference to any features of the offspring distribution besides its mean, and figure 5 confirms that the prediction holds for all our sample contact distributions. Differences among contact distributions are primarily due to our comparison at fixed *T*_*i*_ rather than fixed *R*_0,*i*_.

More generally, Serra & Haccou (2007) give a formal derivation for probability of ‘escape’ (or emergence), showing that (in our notation)

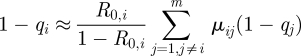

under quite general conditions, including the scenarios considered by Antia *et al.* (2003) and Iwasa *et al.* (2003, 2004). Specifically, this approximation is valid under the following assumptions.

— Total offspring distribution for each type is arbitrary, provided its variance is finite. The mean of the distribution for type *i* is *R*_0,*i*_. Mutations occur independently among the offspring.
— There is a single ‘escape’ type (which we denote *m*) having *R*_0,*m*_ > 1; all other types *i* ≠ *m* have *R*_0,*i*_ < 1.
— There are no mutations away from the escape type; thus, *q*_*m*_ can be computed independently and substituted into the above approximation for *q*_*i*_, *i* ≠ *m*. However, all other mutations among types are allowed.
— All mutation probabilities *μ*_*ij*_, *i* ≠ *j*, are of *O*(*u*).

Under these conditions, the above approximation has error of *O*(*u*^2^). However, the neglected error term increases as *R*_0,*i*_ → 1, implying that the approximation will break down if strain fitness approaches the critical threshold. Iwasa *et al.* (2004) present approximations valid for various choices of *R*_0,*i*_, including the near-critical case, though only for a specific offspring distribution.

**Figure 6.**
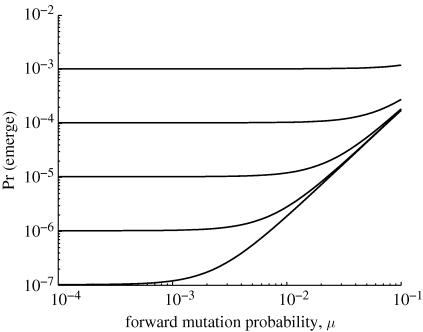
With point mutation (probability *μ*) and recombination (probability *ρ*), probability of emergence (1 − *q*_1_) scales as *O*(*μ*^*m*−1^) + *O*(*ρ*). Each curve corresponds to a different value of *ρ*, spaced logarithmically from 10^−6^ (bottom) to 10^−2^ (top). Contact distribution is Poisson with mean 30; *m* = 3; and 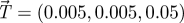.

As an example, consider our ‘mutation and recombination’ scheme. Either by applying an Antia-like argument to this specific scenario or by substituting mutation probabilities into Serra and Haccou's result, we can show that the probability of emergence starting from a type 1 individual (1 − *q*_1_) scales as *O*(*μ*^*m*−1^) + *O*(*ρ*), where *μ* is the probability of one-step forward ‘point mutation’ and *ρ* is the probability of jump-to-*m* ‘recombination’. Figure 6 supports this prediction for an example with *m* = 3 strains, illustrating the relative contributions of the different adaptive pathways. When *ρ* ≫ *μ*^2^, the probability of emergence is almost constant in *μ* and scales approximately linearly in *ρ*. When *ρ* ≪ *μ*^2^, the probability of emergence is almost constant in *ρ* and scales proportionally to *μ*^2^.

Despite the availability of these useful analytical results, there is still a place for numerical solutions when looking for subtle differences among cases (e.g. different contact distributions) or where the assumptions underlying these approximations are broken or pushed to their limits (e.g. relatively large mutation probabilities), as we will encounter in the next subsection.

### 3.6. Impact of increasing overall mutation rate

The same biological mechanisms (e.g. nucleotide substitution rate, error-correcting mechanisms) contribute to all mutation rates, not only those for beneficial mutations. Given this constraint, it is not immediately clear whether pathogens with the highest mutation rates will have the largest or smallest probability of emergence. We explore this question for two mutational schemes in which deleterious steps are possible: one-step reversible mutation and hub-and-spoke irreversible mutation.

**Figure 7.**
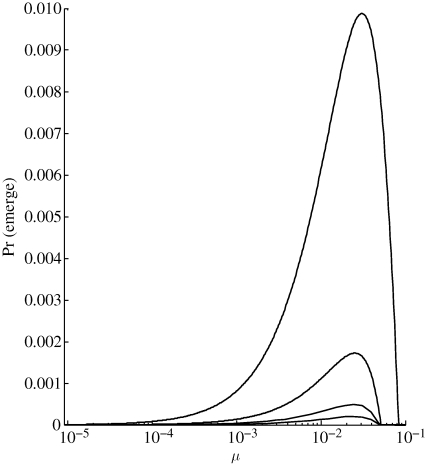
Probability of emergence (1 − *q*_1_) versus forward mutation probability (*μ*) in one-step reversible scheme, where reverse mutation probability *ν* is equal to *μ*. Contact distribution is Poisson with mean 30 and *m* = 2. *T*_1_ is set to (bottom to top curves) 0.005, 0.01, 0.02 or 0.03; while *T*_2_ is fixed at 0.035, slightly above the critical threshold (i.e. 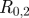 slightly greater than 1).

For simplicity, with one-step reversible mutation, we use only two strains (implying that linear and hub-and-spoke schemes are equivalent), the poorly adapted strain 1 and the well-adapted strain 2. Mutation from 1 to 2 occurs with probability *μ*, and from 2 to 1 with probability *ν*, which we scale proportionally to *μ*. Results demonstrate a non-monotonic relationship between mutation rate and probability of emergence (figure 7). The probability of emergence increases with mutation rate over a wide range, before abruptly crashing to zero. This occurs because the branching process becomes subcritical once reverse mutation rate is too high, even when forward mutation rate is similarly high (§3.3). Note, however, that the crash does not occur if *T*_2_ is well above the critical threshold.

**Figure 8.**
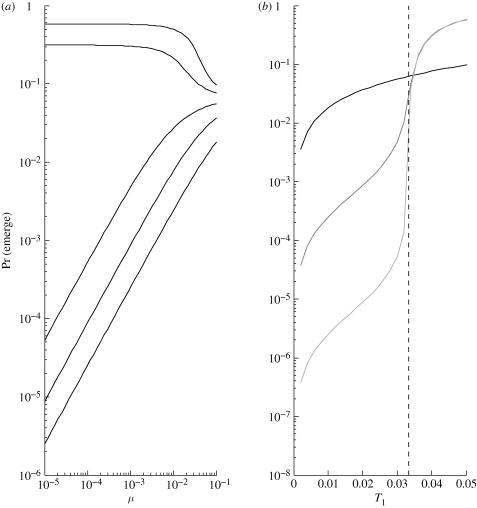
Probability of emergence (1 − *q*_1_) in the irreversible hub-and-spoke mutation scheme. Contact distribution is Poisson with mean 30. There are 10 strains, with mutation occurring with equal probability *μ* from strain 1 to each other strain. *T*_*m*_ = 0.05 while *T*_*i*_ = 0 for *i* = 2, … , *m* − 1. (*a*) We plot versus *μ* for various values of *T*_1_ ranging from 0.01 (bottom) to 0.05 (top), in increments of 0.01. (*b*) We plot versus *T*_1_ for various values of *μ*: 10^−5^ (light grey), 10^−3^ (dark grey) and 10^−1^ (black). The vertical dashed line indicates the critical threshold at which *R*_0,1_ reaches one.

With an irreversible hub-and-spoke mutation scheme, we assume that the initial strain is equally likely to mutate to any other, but only one mutational pathway is beneficial. As an extreme case, we set transmissibility of all deleterious strains to be zero. Figure 8 plots the probability of emergence versus *T*_1_ for various mutation probabilities. When *T*_1_ is well below the critical threshold (at which *R*_0,1_ reaches 1), emergence probability increases linearly with mutation probability, as we would predict from analytical approximations (§3.5). Although this figure plots results for *m* = 10 strains, a virtually identical increase in emergence probability versus *μ* at fixed small *T*_1_ was observed for *m* = 2, 3, 5 as well (results not shown). This indicates that regardless of how high the risk of deleterious steps, a pathogen that is guaranteed to go extinct unless it acquires a beneficial mutation (evolves to strain *m*) is always better off having a large mutation rate. Near the critical threshold, emergence probability shows a sharp jump; once strain 1 is sufficiently fit to escape extinction without further evolution, mutation probability makes only a small difference in results. Shortly after this point, a very high mutation probability becomes detrimental to the pathogen, even while strain 1 is not as well adapted as strain *m*. The risk of mutating to a poorly adapted strain now outweighs the potential benefit of mutating to a better adapted strain. Adding reverse mutation at a probability equal to that of forward mutation makes negligible difference to quantitative results (results not shown), as also observed with linear mutation schemes where *R*_0,*m*_ is not too close to one. We might suspect that increasing reverse mutation probability independently of forward mutation probability could provide a benefit to the pathogen if the transmissibility of deleterious strains is non-zero, such that mutation could ‘rescue’ the pathogen by a return to strain 1 (the hub). However, this does not appear to be the case within tested parameter ranges (results not shown): increasing reverse mutation probability decreases probability of emergence, though only marginally when *R*_0,*m*_ is well above one.

## 4. Discussion

We have linked phenomenological and contact network-based approaches to modelling disease spread as a branching process, clarifying how they may be viewed within a common framework. By explicitly considering the number of contacts along with a model of how transmission occurs to these contacts, a network-based model offers more detailed insights into factors contributing to pathogen fitness and probability of emergence. Such a model lends itself to comparison of the relative impact of various public health interventions, which may act by reducing either number of contacts or probability of transmission per contact (Meyers *et al.* 2005; Brauer 2008).

Expressing the basic reproductive number as *R*_0_ = (*g*′(1)(1 + *c*^2^) − 1)*T*—where *g*′(1) is the mean of the contact distribution, *c* is its coefficient of variation and *T* is transmissibility—highlights the contributions of both ecological factors (contact structure) and epidemiological factors (transmissibility) to pathogen fitness. That is, a pathogen's fitness depends on the particular host population in which it circulates, an observation that has been made more generally for pathogen fitness measures (Antolin 2008). Similarly, in the multi-type process, we can express the mean matrix as *M* = (*g*′(1)(1 + *c*^2^) − 1)diag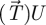, where 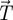 is the vector of strain transmissibilities and *U* is the mutation matrix. These expressions make it clear that for fixed transmissibilities, mean number of contacts and mutation scheme, maximizing pathogen fitness is equivalent to maximizing the variance of the host population's contact distribution; this agrees with the general observation that increasing heterogeneity among hosts increases *R*_0_ (Antolin 2008). Furthermore, in the multi-type process, we see that criticality is independent of mutation probabilities if mutation is uni-directional; however, if mutation is bi-directional then this can push the process into a subcritical range (figure 3).

We have argued that the comparison of emergence probability across contact distributions should be made at given pathogen transmissibility, rather than at given pathogen fitness (*R*_0_). This comparison more easily lends itself to questions about the sort of host population contact structure that is associated with greatest risk of disease emergence. In contrast, holding *R*_0_ fixed in comparisons requires that we simultaneously alter pathogen transmissibility when altering the contact distribution, thereby confounding these two factors.

### 4.1. What are the most risky contact structures?

The contact distribution associated with highest risk of emergence is not always the same, but rather depends on the transmissibility, i.e. the particular pathogen involved. Especially large differences among contact distributions occur near the critical threshold for the branching process. Recall figure 2*b*: at a transmissibility of 0.03, probability of emergence ranges from 0 to approximately 0.7, depending on contact distribution, even when all distributions have the same mean. This suggests that certain pathogens, having a transmissibility near the critical threshold, will be able to cause an epidemic in some host populations, but not others.

Although previous authors have often found that increased population heterogeneity increases the probability of extinction (e.g. Caraco *et al.* 1998; Lloyd-Smith *et al.* 2005), in phenomenological models this heterogeneity is necessarily introduced directly to the offspring distribution. In contrast, the heterogeneity we introduce through contacts in our more mechanistic model has the counteracting effect of raising *R*_0_, as the chance of getting the disease is correlated with the number of opportunities to pass it on. (Recall the distinction between degree and excess degree distribution.) Through comparison of contact distributions at given transmissibility, we found that heterogeneity can in fact increase the risk of emergence over a significant range.

### 4.2. What are the most risky mutational processes?

Probability of emergence is highly sensitive to the mutational pathway(s) by which the pathogen may possibly evolve (figure 4). Provided we are not too close to the critical threshold, reverse mutation has very limited impact on this probability and may reasonably be neglected. On the other hand, the possibility of taking large adaptive steps through either simultaneous point mutations or another mechanism of genetic change can have a dramatic impact. Given the finding on simultaneous mutations, it may be worth including higher order terms in other mutation schemes as well. As in the case of contact distribution, however, the ‘most risky’ mutational pathway depends on the pathogen's initial transmissibility. Given a mutational scheme, probability of emergence is also sensitive to mutation probabilities and number of intermediate strains in an analytically predictable manner (figures 5 and 6).

We also considered more realistic situations where deleterious as well as beneficial evolutionary steps are available to the pathogen, either by reverse mutation or by including multiple pathways in strain space. Here, we constrained all types of mutations to occur with proportional probabilities, based on some overall mutation rate of the pathogen. We found that, when the pathogen was initially poorly adapted, increasing overall mutation rate was usually beneficial to an emerging pathogen (i.e. riskier to the host). On the other hand, as the pathogen's initial fitness increases beyond the critical threshold where survival without adaptation is possible, the risk of deleterious mutations comes to outweigh the benefit of potential mutation to a more fit strain, and a high mutation rate becomes a liability (figure 8). Thus, our results largely provide support for the hypothesis that the statistically higher propensity of RNA viruses to emerge is due to their high mutation rate (Cleaveland *et al.* 2001; Woolhouse *et al.* 2005). However, an exception occurs with pathogens for which the fully adapted strain has a basic reproductive number only slightly above one. In this situation, when allowing for reverse mutation, we observed another phenomenon in which emergence probability has a non-monotonic relationship with overall mutation rate (figure 7). If this rate passes a critical point, excessive back mutation to poorly adapted strains implies that the pathogen can no longer sustain itself. This result parallels the phenomenon of ‘error catastrophe’ observed in viral quasi-species models, which offer a theoretical explanation for experimental success in inducing lethal mutagenesis in viruses (Eigen 2002; Anderson *et al.* 2004; Clementi 2008). Although the required ‘mutation’ rate may at first appear unrealistically high, it is important to remember that it does not represent the probability of point mutation during a single replication event, but actually incorporates all factors involved in strain conversion, including genetic changes arising during multiple rounds of replication and within-host strain competition. Taken together, our results suggest that any attempt to alter a pathogen's mutation rate for host benefit should be undertaken with careful consideration of the pathogen's initial fitness and the availability of mutations, both beneficial and deleterious. An attempt to induce lethal mutagenesis seems liable to backfire if the pathogen has significant adaptive potential, as in the case of many emerging pathogens.

### 4.3. Future directions

We have modelled the spread of an emerging pathogen following its introduction to a new host species. This model does not incorporate the dynamics of the introduction itself (or possibly multiple introductions), such as interspecific interactions (Woolhouse *et al.* 2005; Day *et al.* 2006; Kuiken *et al.* 2006; Woolhouse & Gaunt 2007; Lloyd-Smith *et al.* 2009). Indeed, although a better understanding of the dynamics of cross-species transmission is likely to be important in dealing with emerging diseases, few models to date have considered multiple species and phases in cross-over (Lloyd-Smith *et al.* 2009). (However, see Day *et al.* (2006) and Reluga *et al.* (2007) for models incorporating ongoing interactions with animal reservoirs.) Putting our model in a broader context could elucidate the impact of additional risk factors such as pathogen–host range (Cleaveland *et al.* 2001; Taylor *et al.* 2001).

Within the context of transmission in a single-host population, there are several common but potentially significant limitations of our modelling approach. Given the importance of contact structure that we and others have predicted, consideration of more realistic networks may be an important next step. Using a branching process neglects higher order network features, such as loops that persist even in large populations, thus implicitly assuming that the availability of susceptibles is not limited by local saturation. Clustering of contacts is expected to limit disease spread owing to reduction in the availability of susceptibles (Keeling & Eames 2005), and including features such as triangles in networks may have a dramatic impact in decreasing the probability of a large-scale epidemic (Trapman 2007). Thus, we expect the branching process approach to provide an upper bound on the risk of emergence. On the other hand, a recent analysis addressing some apparent inconsistencies in the literature suggests that the impact of clustering on the probability of an epidemic is in fact negligible unless degree is typically small or host-based heterogeneities are large (Miller 2009), providing some support for conclusions drawn from a branching process approach. While some authors (e.g. Ball & Neal 2002; Trapman 2007; Miller 2009) have made progress in modelling stochastic single-strain disease spread with more complicated contact structures, to our knowledge such approaches have not yet been extended to multiple strains.

A second major assumption in our model is that hosts are homogeneous in terms of their epidemiological characteristics. That is, the same transmissibility applies to every host infected with a given strain of pathogen. However, host-based factors are predicted to play a role in emergence risk (Woolhouse *et al.* 2005; Woolhouse & Gaunt 2007), suggesting value in a more realistic model. This would incorporate variability in host characteristics contributing to transmissibility, such as infectivity and susceptibility (Yates *et al.* 2006; Miller 2007). Analysis in the single-strain case predicts that such heterogeneities reduce the probability of an epidemic (Miller 2007, 2009).

Furthermore, both the level of transmissibility (*T*) and the strain conversion process have been treated only phenomenologically in our present between-host model. We (and other authors) have implicitly assumed that any conversion of strains within an individual is instantaneous, with no possibility for co-infection. We have also ignored variation in the precise number of pathogen copies in the body. Realistically, once a mutation arises in a host there will be some dynamical process leading to fixation or loss over time, with coexistence of strains at least temporarily. Both André & Day (2005) and Handel *et al.* (2006) also raise this issue, the latter suggesting that the difficulty of obtaining direct estimates of parameters in the between-host model (e.g. *R*_0_ and conversion rates) supports a move to modelling at the within-host level.

An important avenue for future work will thus be to develop an explicit model of within-host processes, and then to link the within- and between-host scales. In recent years, a number of authors have developed such ‘nested models’ (reviewed in Mideo *et al.* 2008); however, these are typically deterministic, whereas we suggest that stochasticity may be important at both levels. A pathogen may then increase its fitness via multiple routes (Antolin 2008), with potential for conflicting selection at different scales (Gilchrist & Coombs 2006; Coombs *et al.* 2007). We anticipate that antagonistic selection at the within- versus between-host scales would tend to reduce the probability of population-level emergence, as the more transmissible strain (better able to avoid extinction between hosts) is thwarted by dominance of other strain(s) within the host. By extending our model to the within-host scale, we can address these sorts of trade-offs in greater detail, offering further perspectives on risk factors contributing to evolutionary emergence.

## Appendix A. Correspondence of approaches (single-strain models)

The parallels and subtle differences between the phenomenological and network-based approaches suggest several questions regarding when models are equivalent and whether one may be recovered as a special case of another. We explore these questions in the following subsections.

### A.1. Can g*(*z*)* be recovered from G*(*z*)*?

Not all models deal with generation 0 infectives, or we may only have information for ‘typical’ (later-generation) infectives. To connect such cases to a contact network framework, we may ask: given the excess degree distribution, *G*(*z*), can we work backwards to determine the degree distribution, *g*(*z*)?

We have the relationship

Integrating both sides yields

Thus, given only *G*(*z*), *g*(*z*) is under-determined: once a disease is being passed among those who are connected to the contact network, we have no way of knowing how many additional individuals are in the population but have no contacts at all. To determine *g*(*z*), we must impose an additional assumption. Note that the properties of a PGF imply *g*(1) = 1; thus, we have the relationship

Thus, we can find *g*(*z*) if we specify either *p*_0_, the probability of having zero contacts, or *g*′(1), the mean number of contacts. We can calculate the other quantity from the above relationship, subject to the restriction that *p*_0_ ∈ [0,1] and hence *g*′(1) ∈ [0,1/∫_0_^1^ *G*(*s*) d*s*].

### A.2. Under what conditions is g*(*z*)* = G*(*z*)*?

Phenomenological models do not typically distinguish between generation 0 and later-generation infectives. In what case(s) will this perspective be exactly equivalent to a network-based model? That is, suppose every infective, whether in the initial or later generations, has exactly the same distribution of still-susceptible contacts: *g*(*z*) = *G*(*z*). What restrictions does this impose on the possible distribution of contacts?

Since *G*(*z*) = *g*′(*z*)/*g*′(1), the condition that *g*(*z*) = *G*(*z*) leads to the ordinary differential equation

which, using the constraint that *g*(1) = 1, has the unique solution

Since a PGF uniquely identifies a distribution, we can deduce that the contact distribution is Poisson, with only the mean *g*′(1) left to our discretion. Thus, the distinction between generations of infectives is in fact irrelevant in many phenomenological models, which specify a Poisson distribution. Note that if we only require that the number of still-susceptible contacts should have the same *type* of distribution in each generation, but not necessarily with the same parameter(s), then other types of distributions are possible: for instance, a negative binomial degree distribution with mean *λ* and dispersion *β* leads to a negative binomial excess degree distribution with mean *λ*(*β* + 1)/*β* and dispersion *β* + 1.

### A.3. Can G*(*z*)* be recovered from *Γ**(*s*)*?

Recall that authors taking phenomenological approaches (e.g. Antia *et al.* 2003; Lloyd-Smith *et al.* 2005) specify only the distribution of *infectious* contacts. Once we separate contact structure from transmissibility in a network-based approach (as in Brauer 2008), is there more than one distribution of contacts that will achieve the same distribution of infectious contacts? For consistency among models, in the remainder of this section, we will treat all infectives alike, and work only with *G*(*z*) and *Γ*(*s*). The same results apply if we replace *G*(*z*) with *g*(*z*) and *Γ*(*s*) with *γ*(*s*).

Given a distribution *Γ*(*s*) for number of infectious contacts, we can always obtain a distribution *G*(*z*) for number of still-susceptible contacts that satisfies *Γ*(*s*) = *G*(1 − *T* + *Ts*), trivially, by taking *T* = 1 and *G*(*z*) ≡ *Γ*(*z*). More generally, for arbitrary *T*, we require that *G*(1 − *T* + *Ts*) = *Γ*(*s*), if *G*(·) exists for this choice of *Γ*(·). Rearranging *z* = 1 − *T* + *Ts* yields *s* = (*z* − (1 − *T*))/*T*. Thus, *G*(*z*) = *Γ*((*z* − (1 − *T*))/*T*) if *G*(*z*) exists; that is, if this expression yields a valid PGF. Taking *z* ∈ [0,1] as we would usually evaluate a PGF leads to *s* = (*z* − (1 − *T*))/*T* ∈ [−(1 − *T*)/*T*, 1], and *Γ*(*s*) may not be a valid PGF on this range. Taking *Γ*(*s*) to be, say, binomial or negative binomial leads to a valid PGF for *G*(*z*), for any *T* ∈ [0,1]. But taking *Γ*(*s*) = *s*^*N*^ (exactly *N* infections transmitted by every infective) only gives a valid PGF if *T* = 1, not surprisingly. These examples illustrate that some, but not all, distributions of infectious transmissions can be traced back to contact distributions for arbitrary *T*. This limitation is to be expected, given that we have constrained the number of transmissions from an individual to be distributed binomially with total number of ‘trials’ equal to the number of contacts. Precise conditions on *Γ*(*s*) yielding a valid contact distribution *G*(*z*) in this or other models of transmission is an interesting mathematical question, but lies beyond the scope of the present work.

## Appendix B. Detailed descriptions of contact distributions and mutational schemes

### B.1. Contact distributions

The following contact distributions are used in our numerical results. In all cases, *λ* denotes the mean of the distribution. Tables 1 and 2 summarize these distributions with the corresponding PGFs and expressions for *R*_0_.

**Table 1.** Names and associated PGFs of distributions used in numerical results. Listed parameters are the mean for deterministic and Poisson; number of trials and probability of success for binomial; mean and dispersion for negative binomial; proportion of each type and mean for each type for mixed deterministic and mixed Poisson; and proportion of each type, number of trials for each type, and probability of success for mixed binomial. The PGF for a Poisson distribution is given by Brauer (2008) and the PGF for a negative binomial distribution is given by Lloyd-Smith *et al.* (2005).

contact (degree) distribution	excess degree distribution	offspring distribution (single strain)
deterministic(*λ*)	deterministic(*λ* − 1)	binomial(*λ* − 1, *T*)
*g*(*z*) = *z*^*λ*^	*G*(*z*) = *z*^*λ*−1^	*Γ*(*s*) = (1 − *T* + *Ts*)^*λ*−1^
Poisson(*λ*)	Poisson(*λ*)	Poisson(*λ**T*)
*g*(*z*) = exp(*λ*(*z* − 1))	*G*(*z*) = exp(*λ*(*z* − 1))	*Γ*(*s*) = exp(*λ**T*(*s* − 1))
negative binomial(*λ*, *β*)	negative binomial(*λ*(*β* + 1)/*β*, *β* + 1)	negative binomial(*λ**T*(*β* + 1)/*β*, *β* + 1)
*g*(*z*) = (1 + (*λ*/*β*)(1 − *z*))^−*β*^	*G*(*z*) = (1 + (*λ*/*β*)(1 − *z*))^−(*β*+1)^	*Γ*(*s*) = (1 + (*λ**T*/*β*)(1 − *s*))^−(*β*+1)^
mixed deterministic(, )	mixed deterministic(, )	mixed binomial(, , *T*)
*g*(*z*) = ∑_*k*=1_^*n*^ *p*_*k*_ *z*^*λ*_*k*_^	*G*(*z*) = (1/*λ*) ∑_*k*=1_^*n*^ *p*_*k*_*λ*_*k*_ *z*^*λ*_*k*_−1^	*Γ*(*s*) = (1/*λ*) ∑_*k*=1_^*n*^ *p*_*k*_ *λ*_*k*_ (1 − *T* + *Ts*)^*λ*_*k*_−1^
mixed Poisson(, )	mixed Poisson(, )	mixed Poisson(, )
*g*(*z*) = ∑_*k*=1_^*n*^ *p*_*k*_ exp(*λ*_*k*_ (*z* − 1))	*G*(*z*) = (1/*λ*) ∑_*k*=1_^*n*^ *p*_*k*_*λ*_*k*_ exp(*λ*_*k*_ (*z* − 1))	*Γ*(*s*) = (1/*λ*) ∑_*k*=1_^*n*^ *p*_*k*_ *λ*_*k*_ exp(*λ*_*k*_ *T*(*s* − 1))

**Table 2.** Contact distributions used in numerical results, along with their variance and the expression for the basic reproductive number *R*_0_ for the single-strain model in each case.

contact (degree) distribution	variance	*R*_0_
deterministic(*λ*)	0	(*λ* − 1)*T*
Poisson(*λ*)	*λ*	*λ**T*
negative binomial(*λ*, *β*)	*λ*(1 + *λ*/*β*)	*λ**T*(*β* + 1)/*β*
mixed deterministic(, )	∑_*k*=1_^*n*^ *p*_*k*_ (*λ*_*k*_ − *λ*)^2^	(1/*λ*) ∑_*k*=1_^*n*^ *p*_*k*_ *λ*_*k*_ (*λ*_*k*_ − 1)*T*
mixed Poisson(, )	*λ* + ∑_*k*=1_^*n*^ *p*_*k*_ (*λ*_*k*_ − *λ*)^2^	(1/*λ*) ∑_*k*=1_^*n*^ *p*_*k*_*λ*_*k*_^2^ *T*

*Deterministic.* Every individual has a fixed number of contacts. This leads to a binomial offspring distribution.

*Poisson.* This distribution is appropriate ‘[i]f contacts between members of the population are random, corresponding to the assumption of mass action in the transmission of disease’ (Brauer 2008, p. 142). The degree and excess degree distributions are the same. Using a Poisson-distributed number of contacts also leads to a Poisson-distributed number of *infectious* contacts (offspring distribution), as used in several phenomenological models (e.g. Antia *et al.* 2003; Yates *et al.* 2006; Reluga *et al.* 2007). In fact, since a Poisson distribution is fully determined by its mean, disease outbreak properties in this situation are fully determined by *R*_0_ (or *R*_0,*i*_ for multiple strains), regardless of whether *R*_0_ is influenced through contacts or transmissibility. However, for other distributions, the parameters involved in the model do not appear only in the combination *R*_0_; that is, for a fixed choice of *R*_0_, the probability of emergence still varies depending on the chosen parameters. Here, the separation of ecological and epidemiological influences is significant.

*Negative binomial.* This type of distribution arises in Lloyd-Smith *et al.* (2005) by taking a Poisson distribution with mean drawn from a Gamma distribution. We might interpret this distribution as arising from an infective making contacts at a constant rate (i.e. at points of a Poisson process) over a Gamma-distributed duration of infection. Variance decreases as dispersion (*β*) increases; in the limit as *β* → ∞, we get a Poisson distribution.

*Mixed distributions.* This choice of distribution is motivated by the existence in the host population of ‘superspreaders’, who contribute an unusually large number of transmission events (Lloyd-Smith *et al.* 2005; Meyers *et al.* 2005; Day *et al.* 2006; Yates *et al.* 2006; Brauer 2008). We introduce this heterogeneity through number of contacts, an ecological effect, similarly to examples in Brauer (2008). Another possibility is that superspreading is due to higher-than-normal transmissibility among certain members of the population (Yates *et al.* 2006), an epidemiological effect that might be incorporated through an extension to our model (§4.3). While phenomenological models directly specifying the distribution of infectious contacts would not distinguish the two cases, which both simply raise *R*_0_ (e.g. Lloyd-Smith *et al.* 2005), these situations may lead to very different results (Meyers *et al.* 2005). Some authors distinguish the two cases by labelling hosts with a large number of contacts ‘superspreaders’, and those with high transmissibility ‘supershedders’ (Meyers *et al.* 2005).

In general, a mixed distribution specifies *n* types of individuals, in proportions *p*_1_, … , *p*_*n*_. Each type has a characteristic distribution of contacts, which we take to be either deterministic (the *k*th type has exactly *λ*_*k*_ contacts) or Poisson (the *k*th type has a Poisson(*λ*_*k*_) distribution of contacts), where ∑_*k*=1_^*n*^ *p*_*k*_*λ*_*k*_ = *λ*. These cases lead to a mixed binomial or a mixed Poisson offspring distribution, respectively. For our numerical examples, we have taken *n* = 2, representing a less-frequent class of superspreaders among more-frequent ‘normal’ spreaders.

### B.2. Mutational schemes

Schemes are numbered and interpreted as in §2.2. Each is characterized by the mutation matrix *U* = [*μ*_*ij*_], where *μ*_*ij*_ is the probability that an infection transmitted by a type *i* is of type *j*. We denote the probability of one-step forward point mutation by *μ*, of one-step reverse point mutation by *ν* and of jump-to-strain-*m* genetic change (e.g. recombination) by *ρ*.

*One-step irreversible:*

*Multi-step irreversible:* following Gokhale *et al.* (2009),

*Interchangeable and irreversible:* following Gokhale *et al.* (2009),

*Point mutation and recombination:*

*One-step reversible:*

*Hub-and-spoke, irreversible:*

*Hub-and-spoke, reversible:*

## Appendix C. Derivation of PGFs for the multi-type model

Here, we present a derivation of the PGFs used in our network-based multi-type model. First consider the PGF for number of infections of each type, , transmitted by a type  infective, given  susceptible contacts. The number of each type of transmission (including ‘failed’ transmissions) has a multinomial distribution with *d* independent ‘trials’; possible outcomes are transmission of type *j* (probability *T*_*i*_*μ*_*ij*_), for some *j* = 1, …, *m*, or no transmission (probability 1 − *T*_*i*_). Thus,

where at the final step we have used the multinomial theorem to rewrite the summation.

We can then derive the offspring distribution by considering the distribution of *d*. The appropriate PGF depends on the generation:

Finally, consider the overall probability of extinction starting from an initial infective of type *i*, given the probability of extinction starting from a later-generation infective of each type (). Let *D* be the number of contacts of the initial case and let  be the number of infectives of each type in generation 1. Then, using the independence of the branching processes started in generation 1, we have

## Appendix D. Technical results for multi-type branching processes

In this section, we present more detailed technical results relevant to the methods applied to our multi-type branching process. As in the main text, we denote by  the vector of type-specific offspring distribution PGFs. First, we make the reasonable assumption that our process is never singular. Singular processes are those in which every individual has exactly one offspring (Harris 1963), and are often excluded from analyses.

Results for multi-type branching processes are usually stated for indecomposable processes, in which ‘each type of individual eventually may have progeny of any other type’ (Haccou *et al.* 2005, p. 26), also known as irreducible processes (Mode 1971). Such a process can be identified from the expectation matrix, *M*: the process is indecomposable if, for every pair of types (*i,j*), there exists a positive integer *n* such that *M*^*n*^(*i,j*) > 0; or equivalently, there is a non-zero probability of producing at least one type *j* in generation *n*, given that the process starts with one type *i* (Mode 1971; Haccou *et al.* 2005). If *n* is independent of the types (*i,j*), the process is called positively regular (Mode 1971). Only periodic processes are indecomposable, but fail to be positively regular (Mode 1971); however, a periodic process can be represented by a non-periodic process through a transformation of time scale (Haccou *et al.* 2005). Thus, from here on, we take indecomposable to mean positively regular. In our model, whenever mutation is reversible and all types are transmissible, we have an indecomposable process.

On the other hand, we may be dealing with a decomposable process, in which there are ‘distinct groups of types that do not produce types in other groups’ (Haccou *et al.* 2005, p. 27). In our model, whenever mutation is irreversible, the mutation matrix *U* is upper triangular; hence, the expectation matrix *M* and any positive power *M*^*n*^ are also upper triangular and we have a decomposable process. We also encounter decomposable processes whenever there are non-transmitting types, leading to an all-zero row in *M*.

We say that extinction of the process occurs when the population of every type reaches zero at some generation. Analogously to the single-type case, there is a threshold theorem for multi-type processes stating when non-extinction is possible; now, the threshold parameter is the dominant eigenvalue *ρ* of the expectation matrix *M*. In the indecomposable case, extinction occurs with probability one from any starting type if and only if *ρ* ≤ 1 (Harris 1963; Mode 1971; Athreya & Ney 1972; Allen 2003; Haccou *et al.* 2005). In the decomposable case, we must consider classes of types that communicate, i.e. any type within a class can give rise to any other type in the class after some number of generations (Harris 1963; Mode 1971). Then, provided there is no type that produces in the next generation exactly one offspring in its class with probability one, the threshold theorem continues to hold in the decomposable case (Harris 1963; Mode 1971). We will assume that this condition, which is essentially an extension of the definition of non-singularity (Mode 1971), always holds in processes we consider.

Applying this theorem to our model, clearly the possibility of non-extinction requires that some type is supercritical in isolation, i.e. *R*_0,*i*_ > 1 for some *i*. When mutation is irreversible, the eigenvalues of *M* are simply given by the entries on the main diagonal, and assuming that the final strain *m* is best adapted, the dominant eigenvalue is *R*_0,*m*_. Thus, the process is guaranteed to go extinct from every initial type if and only if *R*_0,*m*_ ≤ 1. Otherwise, there is a positive probability that the process does not go extinct, at least from some initial type. In fact, any initial type will suffice, provided the probability of forward mutation is positive and every strain is transmissible. For reversible mutation, again assuming strain *m* is best adapted, having *R*_0,*m*_ > 1 is a necessary, but not sufficient, condition to obtain a positive probability of non-extinction (cf. figure 3). Finally, if any type is non-transmissible (as we sometimes consider with hub-and-spoke schemes), clearly a process initiated by such a type is guaranteed to go extinct. Starting from any other type, we can simply ignore any production of non-transmissible types to define a process fitting into one of the aforementioned cases.

Let *q*_*i*_ denote the extinction probability of a multi-type branching process initiated by one type *i* individual. It is a well-known result for an indecomposable process that  is a fixed point of the vector equation , i.e. ,  (Harris 1963; Athreya & Ney 1972; Kimmel & Axelrod 2002; Allen 2003; Haccou *et al.* 2005). However, the argument for reaching this conclusion in fact applies to *any* branching process (Mode 1971); some further deductions depend on irreducibility. First note that  (the vector of length *m* containing all ones) is always a fixed point of ; there may be other solutions too.

For an indecomposable process,  is the smallest non-negative fixed point of : if *ρ* ≤ 1, then , as guaranteed by the threshold theorem; while if *ρ* > 1 (the supercritical case), 0 ≤ *q*_*i*_ < 1,  (Harris 1963; Mode 1971; Athreya & Ney 1972; Kimmel & Axelrod 2002; Allen 2003; Haccou *et al.* 2005). Furthermore, the only solutions to the fixed-point equation with , , are  and , and fixed-point iteration beginning with any  (0 ≤ *s*_*i*_ < 1, ) converges to  (Harris 1963; Mode 1971; Athreya & Ney 1972). This suggests a simple way to compute extinction probabilities to arbitrary accuracy, and is indeed the method we use for our numerical results (§3.3).

For a decomposable process, the threshold theorem again implies that  if *ρ* ≤ 1. However, for a supercritical process (*ρ* > 1), the uniqueness of fixed-point solutions  does not necessarily hold, nor must every *q*_*i*_ be less than one even when . For any situation we consider, we can argue (see below) that our computational method finds the ‘right’ solution  representing the probability that the entire process (i.e. every type) goes extinct; see also Reluga *et al.* (2007) for interpretations of multiple solutions.

We first assume that all types are transmissible, so *M* has no all-zero row. Suppose mutation is irreversible and hence *M* is upper triangular. This implies that  is in fact independent of *s*_*j*_ for all *j* < *i*. Thus, we can solve for any fixed point  by working backwards through types *m*, *m* − 1, … , 1, substituting solutions for those *s*_*i*_ already found and leaving an equation in one variable only. Assuming type *m* is supercritical (*R*_0,*m*_ > 1), according to results for single-type branching processes, *Γ*_*m*_(*s*_*m*_) = *s*_*m*_ has two solutions: *s*_*m*_ = 1 and *s*_*m*_ = *q*_*m*_ < 1 (the extinction probability of the process initiated by a type *m*). We can then solve for *s*_*m*−1_ in each case. *Γ*_*m*−1_(*s*_*m*−1_, 1) is the PGF for the number of type *m* − 1's produced by a type *m* − 1, defining a single-type branching process. In most cases we consider, *R*_0,*m*−1_ < 1, and hence this will be a subcritical branching process, yielding a unique fixed point on [0, 1], *s*_*m*−1_ = 1. Occasionally (e.g. figure 8), we will consider a case where *R*_0,*m*−1_ > 1 and the process may be supercritical; in this case, we obtain two fixed points, *s*_*m*−1_ = 1 and *s*_*m*−1_ = *q̃*_*m*−1_ < 1. On the other hand, *Γ*_*m*−1_(*s*_*m*−1_, *q*_*m*_) is an increasing, concave-up function satisfying *Γ*_*m*−1_(0, *q*_*m*_) > 0 and, assuming *μ*_*m*−1,*m*_ > 0 (i.e. *Γ*_*m*−1_ is not independent of *s*_*m*_), *Γ*_*m*−1_(1, *q*_*m*_) < 1; therefore, there is a unique solution *q*_*m*−1_ < 1 satisfying *Γ*_*m*−1_(*q*_*m*−1_, *q*_*m*_) = *q*_*m*−1_. The argument proceeds identically as we continue to work backwards through types. To summarize, in most cases, we will have *R*_0,*m*_ > 1 and *R*_0,*i*_ < 1 ; this yields two solutions to the vector fixed-point equation:  and , the extinction probability, where *q*_*i*_ < 1, . Furthermore, fixed-point iteration beginning with any  with all components <1 will converge to the latter solution, since iteration of *Γ*_*m*_(*s*_*m*_) converges to *q*_*m*_. In the occasional case that we have more than one supercritical type, there will be more than two solutions, corresponding to different subsets of types persisting in the case of non-extinction (Reluga *et al.* 2007). However, only one solution  will have all components <1, and this can readily be identified as the vector of probabilities that the entire process (every type) goes extinct, based on arguments presented above. Again, this is the solution that will be reached by fixed-point iteration beginning with  such that *s*_*i*_ < 1, , which is the method we apply in computations. In more complicated mutational schemes (not considered here), for instance if certain pathways are reversible and others are not, classes in a decomposable process may consist of more than one type; here we can write *M* in a block triangular form and apply results from Mode (1971) to make similar arguments.

If any type *i* is not transmissible, its corresponding PGF is clearly *Γ*_*i*_ , with unique fixed point *s*_*i*_ = 1. Substituting this solution into all other equations yields PGFs that effectively neglect any production of type *i*, and we can then proceed as described above.

Finally, recall that the probability of extinction starting from a generation 0 infective of type *i*, chosen uniformly at random with respect to degree, is given by *g*(1 − *T*_*i*_ + *T*_*i*_∑_*j*=1_^*m*^ *μ*_*ij*_ *q*_*j*_) ≡ *γ*_*i*_. This probability is less than one if and only if *q*_*j*_ < 1 for some *j* for which *μ*_*ij*_ > 0, simply meaning that extinction is guaranteed in this process if it is guaranteed in the processes initiated by later-generation infectives of types accessible by mutation.

If the branching process escapes extinction, we may be interested in the complement of types present in the long term. In indecomposable processes, all types will persist, and their populations grow asymptotically at a geometric rate *ρ* (Haccou *et al.* 2005). However, this is not necessarily the case in decomposable processes. A simple inductive argument can be used to show that only supercritical types can persist if mutation is irreversible; so, in most cases we consider, ‘emergence’ means that only type *m* is present in the long term. More detailed limit theorems can be found in Mode (1971).

## References

[RSIF20100123C1] Allen L. J. S. 2003 An introduction to stochastic processes with applications to biology. Upper Saddle River, NJ: Pearson Education, Inc.

[RSIF20100123C2] Anderson R. M. May R. M. 1991 Infectious diseases of humans: dynamics and control. Oxford, UK: Oxford University Press.

[RSIF20100123C3] Anderson J. P. Daifuku R. Loeb L. A. 2004 Viral error catastrophe by mutagenic nucleosides. Annu. Rev. Microbiol. 58, 183–205. (10.1146/annurev.micro.58.030603.123649) 15487935

[RSIF20100123C4] André J.-B. Day T. 2005 The effect of disease life history on the evolutionary emergence of novel pathogens. Proc. R. Soc. B 272, 1949–1956. (10.1098/rspb.2005.3170) PMC1559879 16191602

[RSIF20100123C5] Antia R. Regoes R. R. Koella J. C. Bergstrom C. T. 2003 The role of evolution in the emergence of infectious diseases. Nature 426, 658–661. (10.1038/nature02104) 14668863 PMC7095141

[RSIF20100123C6] Antolin M. F. 2008 Unpacking *β*: within-host dynamics and the evolutionary ecology of pathogen transmission. Annu. Rev. Ecol. Evol. Syst. 39, 415–437. (10.1146/annurev/ecolsys.37.091305.110119)

[RSIF20100123C7] Athreya K. B. Ney P. E. 1972 Branching processes. Berlin, Germany: Springer-Verlag.

[RSIF20100123C8] Ball F. Neal P. 2002 A general model for stochastic SIR epidemics with two levels of mixing. Math. Biosci. 180, 73–102. (10.1016/S0025-5564(02)00125-6) 12387917

[RSIF20100123C9] Brauer F. 2008 An introduction to networks in epidemic modeling. In Mathematical epidemiology (eds Brauer F. van den Driessche P. Wu J.), Lecture Notes in Mathematics, no. 1945, pp. 133–146. Berlin, Germany: Springer-Verlag.

[RSIF20100123C10] Caraco T. Duryea M. Gardner G. Maniatty W. Szymanski B. K. 1998 Host spatial heterogeneity and extinction of an SIS epidemic. J. Theor. Biol. 192, 351–361. (10.1006/jtbi.1998.0663) 9650291

[RSIF20100123C11] Cleaveland S. Laurenson M. K. Taylor L. H. 2001 Diseases of humans and their domestic mammals: pathogen characteristics, host range and the risk of emergence. Phil. Trans. R. Soc. Lond. B 356, 991–999. (10.1098/rstb.2001.0889) 11516377 PMC1088494

[RSIF20100123C12] Clementi M. 2008 Perspectives and opportunities for novel antiviral treatments targeting virus fitness. Clin. Microbiol. Infect. 14, 629–631. (10.1111/j.1469-0691.2007.01937.x) 18190573

[RSIF20100123C13] Coombs D. Gilchrist M. A. Ball C. L. 2007 Evaluating the importance of within- and between-host selection pressures on the evolution of chronic pathogens. Theor. Popul. Biol. 72, 576–591. (10.1016/j.tpb.2007.08.005) 17900643

[RSIF20100123C14] Day T. André J.-B. Park A. 2006 The evolutionary emergence of pandemic influenza. Proc. R. Soc. B 273, 2945–2953. (10.1098/rspb.2006.3638) PMC1639509 17015361

[RSIF20100123C15] Dennehy J. J. 2009 Bacteriophages as model organisms for virus emergence research. Trends Microbiol. 17, 450–457. (10.1016/j.tim.2009.07.006) 19765997 PMC7127698

[RSIF20100123C16] Eigen M. 2002 Error catastrophe and antiviral strategy. Proc. Natl Acad. Sci. USA 99, 13 374–13 376 (10.1073/pnas.212514799) PMC129678 12370416

[RSIF20100123C17] Gilchrist M. A. Coombs D. 2006 Evolution of virulence: interdependence, constraints, and selection using nested models. Theor. Popul. Biol. 69, 145–153. (10.1016/j.tpb.2005.07.002) 16198387

[RSIF20100123C18] Gokhale C. S. Iwasa Y. Nowak M. A. Traulsen A. 2009 The pace of evolution across fitness valleys. J. Theor. Biol. 259, 613–620. (10.1016/j.jtbi.2009.04.011) 19394348 PMC2711507

[RSIF20100123C19] Haccou P. Jagers P. Vatutin V. A. 2005 Branching processes: variation, growth, and extinction of populations. Cambridge, UK: Cambridge University Press.

[RSIF20100123C20] Handel A. Regoes R. R. Antia R. 2006 The role of compensatory mutations in the emergence of drug resistance. PLoS Comput. Biol. 2, 1262–1270. (10.1371/journal.pcbi.0020137) PMC1599768 17040124

[RSIF20100123C21] Harris T. E. 1963 The theory of branching processes. Berlin, Germany: Springer-Verlag.

[RSIF20100123C22] Iwasa Y. Michor F. Nowak M. 2003 Evolutionary dynamics of escape from biomedical intervention. Proc. R. Soc. Lond. B 270, 2573–2578. (10.1098/rspb.2003.2539) PMC1691545 14728779

[RSIF20100123C23] Iwasa Y. Michor F. Nowak M. 2004 Evolutionary dynamics of invasion and escape. J. Theor. Biol. 226, 205–214. (10.1016/j.jtbi.2003.08.014) 14643190

[RSIF20100123C24] Keeling M. J. Eames K. T. D. 2005 Networks and epidemic models. J. R. Soc. Interface 2, 295–307. (10.1098/rsif.2005.0051) 16849187 PMC1578276

[RSIF20100123C25] Kimmel M. Axelrod D. E. 2002 Branching processes in biology. New York, NY: Springer-Verlag.

[RSIF20100123C26] Kuiken T. Holmes E. C. McCauley J. Rimmelzwaan G. F. Williams C. S. Grenfell B. T. 2006 Host species barriers to influenza virus infections. Science 312, 394–397. (10.1126/science.1122818) 16627737

[RSIF20100123C27] Lloyd-Smith J. O. Schreiber S. J. Kopp P. E. Getz W. M. 2005 Superspreading and the effect of individual variation on disease emergence. Nature 438, 355–359. (10.1038/nature04153) 16292310 PMC7094981

[RSIF20100123C28] Lloyd-Smith J. O. George D. Pepin K. M. Pitzer V. E. Pulliam J. R. C. Dobson A. P. Hudson P. J. Grenfell B. T. 2009 Epidemic dynamics at the human–animal interface. Science 326, 1362–1367. (10.1126/science.1177345) 19965751 PMC3891603

[RSIF20100123C29] May R. M. Lloyd A. L. 2001 Infection dynamics on scale-free networks. Phys. Rev. E 64, 066112 (10.1103/PhysRevE.64.066112) 11736241

[RSIF20100123C30] Meyers L. A. Pourbohloul B. Newman M. E. J. Skowronski D. M. Brunham R. C. 2005 Network theory and SARS: predicting outbreak diversity. J. Theor. Biol. 232, 71–81. (10.1016/j.jtbi.2004.07.026) 15498594 PMC7094100

[RSIF20100123C31] Mideo N. Alizon S. Day T. 2008 Linking within- and between-host dynamics in the evolutionary epidemiology of infectious diseases. Trends Ecol. Evol. 23, 511–517. (10.1016/j.tree.2008.05.009) 18657880

[RSIF20100123C32] Miller J. C. 2007 Epidemic size and probability in populations with heterogeneous infectivity and susceptibility. Phys. Rev. E 76, 010101 (10.1103/PhysRevE.76.010101) 17677396

[RSIF20100123C33] Miller J. C. 2009 Spread of infectious disease through clustered populations. J. R. Soc. Interface 6, 1121–1134. (10.1098/rsif.2008.0524) 19324673 PMC2817154

[RSIF20100123C34] Mode C. J. 1971 Multitype branching processes: theory and applications. Modern Analytic and Computational Methods in Science and Mathematics, no. 34. New York, NY: American Elsevier Publishing Company, Inc.

[RSIF20100123C35] Reluga T. Meza R. Walton D. B. Galvani A. P. 2007 Reservoir interactions and disease emergence. Theor. Popul. Biol. 72, 400–408. (10.1016/j.tpb.2007.07.001) 17719617 PMC2677105

[RSIF20100123C36] Sagitov S. Serra M. C. 2009 Multitype Bienaymé–Galton–Watson processes escaping extinction. Adv. Appl. Probab. 41, 225–246. (10.1239/aap/1240319583)

[RSIF20100123C37] Serra M. C. Haccou P. 2007 Dynamics of escape mutants. Theor. Popul. Biol. 72, 167–178. (10.1016/j.tpb.2007.01.005) 17350060

[RSIF20100123C38] Taylor L. H. Latham S. M. Woolhouse M. E. J. 2001 Risk factors for human disease emergence. Phil. Trans. R. Soc. Lond. B 356, 983–989. (10.1098/rstb.2001.0888) 11516376 PMC1088493

[RSIF20100123C39] Trapman P. 2007 On analytical approaches to epidemics on networks. Theor. Popul. Biol. 71, 160–173. (10.1016/j.tpb.2006.11.002) 17222879

[RSIF20100123C40] Woolhouse M. Gaunt E. 2007 Ecological origins of novel human pathogens. Crit. Rev. Microbiol. 33, 231–242. (10.1080/10408410701647560) 18033594

[RSIF20100123C41] Woolhouse M. E. J. Haydon D. T. Antia R. 2005 Emerging pathogens: the epidemiology and evolution of species jumps. Trends Ecol. Evol. 20, 238–244. (10.1016/j.tree.2005.02.009) 16701375 PMC7119200

[RSIF20100123C42] Yates A. Antia R. Regoes R. R. 2006 How do pathogen evolution and host heterogeneity interact in disease emergence? Proc. R. Soc. B 273, 3075–3083. (10.1098/rspb.2006.3681) PMC1679899 17015347

